# **α** Cell dysfunction in islets from nondiabetic, glutamic acid decarboxylase autoantibody–positive individuals

**DOI:** 10.1172/JCI156243

**Published:** 2022-06-01

**Authors:** Nicolai M. Doliba, Andrea V. Rozo, Jeffrey Roman, Wei Qin, Daniel Traum, Long Gao, Jinping Liu, Elisabetta Manduchi, Chengyang Liu, Maria L. Golson, Golnaz Vahedi, Ali Naji, Franz M. Matschinsky, Mark A. Atkinson, Alvin C. Powers, Marcela Brissova, Klaus H. Kaestner, Doris A. Stoffers, for the HPAP Consortium

**Affiliations:** 1 Department of Biochemistry and Biophysics,; 2 Institute for Diabetes, Obesity, and Metabolism,; 3 Division of Endocrinology, Diabetes, and Metabolism, Department of Medicine,; 4 Department of Genetics, and; 5 Department of Surgery, Perelman School of Medicine, University of Pennsylvania, Philadelphia, Pennsylvania, USA.; 6 Departments of Pathology, Immunology, and Laboratory Medicine, University of Florida Diabetes Institute, Gainesville, Florida, USA.; 7 Department of Pediatrics, University of Florida Diabetes Institute, College of Medicine, Gainesville, Florida, USA.; 8 Division of Diabetes, Endocrinology, and Metabolism, Department of Medicine, Vanderbilt University, Nashville, Tennessee, USA.; 9 VA Tennessee Valley Healthcare System, Nashville, Tennessee, USA.; 10 The Human Pancreas Analysis Program (HPAP) (RRID:SCR_016202).

**Keywords:** Endocrinology, Autoimmune diseases, Diabetes, Islet cells

## Abstract

**BACKGROUND:**

Multiple islet autoantibodies (AAbs) predict the development of type 1 diabetes (T1D) and hyperglycemia within 10 years. By contrast, T1D develops in only approximately 15% of individuals who are positive for single AAbs (generally against glutamic acid decarboxylase [GADA]); hence, the single GADA^+^ state may represent an early stage of T1D.

**METHODS:**

Here, we functionally, histologically, and molecularly phenotyped human islets from nondiabetic GADA^+^ and T1D donors.

**RESULTS:**

Similar to the few remaining β cells in the T1D islets, GADA^+^ donor islets demonstrated a preserved insulin secretory response. By contrast, α cell glucagon secretion was dysregulated in both GADA^+^ and T1D islets, with impaired glucose suppression of glucagon secretion. Single-cell RNA-Seq of GADA^+^ α cells revealed distinct abnormalities in glycolysis and oxidative phosphorylation pathways and a marked downregulation of cAMP-dependent protein kinase inhibitor β (*PKIB*), providing a molecular basis for the loss of glucose suppression and the increased effect of 3-isobutyl-1-methylxanthine (IBMX) observed in GADA^+^ donor islets.

**CONCLUSION:**

We found that α cell dysfunction was present during the early stages of islet autoimmunity at a time when β cell mass was still normal, raising important questions about the role of early α cell dysfunction in the progression of T1D.

**FUNDING:**

This work was supported by grants from the NIH (3UC4DK112217-01S1, U01DK123594-02, UC4DK112217, UC4DK112232, U01DK123716, and P30 DK019525) and the Vanderbilt Diabetes Research and Training Center (DK20593).

## Graphical abstract

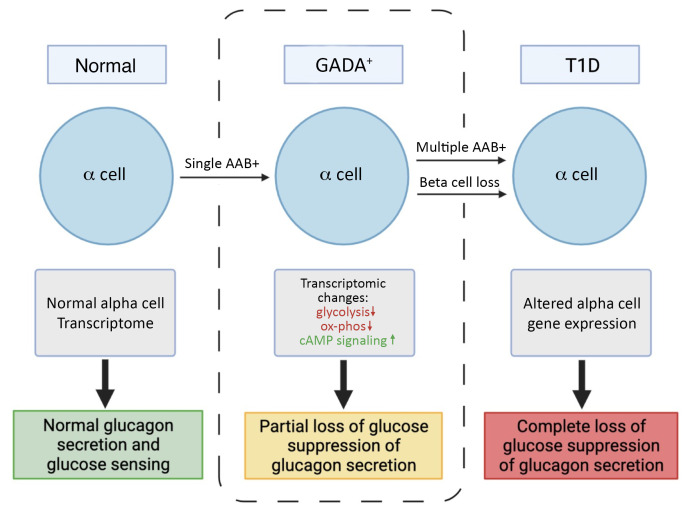

## Introduction

Longitudinal studies have shown that individuals who are at high genetic risk for or have a family history of type 1 diabetes (T1D), who later develop diabetes, progress through several distinct stages prior to the onset of clinical symptoms (1). The presence of islet autoantibodies (AAbs) is currently the best biomarker for the future onset of hyperglycemia in T1D. The presence of 2 or more AAbs confers a 70% risk of developing T1D within 10 years and nearly 100% over the lifetime of the individual. The factors involved in the rate of progression are poorly understood, although a younger age at seroconversion, a higher number of positive AAbs, and higher levels of IAA and IA-2A AAbs have been associated with a more rapid rate of progression to T1D (2–4). In contrast, T1D develops in just 15% of individuals who are single AAb^+^ within 10 years of follow-up, with the anti–glutamic acid decarboxylase AAb (GADA) being by far the most common presenting AAb (2, 3). T1D is typically characterized by the progressive loss of insulin-secreting β cells (1). However, while largely underappreciated, glucagon-secreting α cells are also affected in individuals with T1D and contribute to the pathophysiology of diabetes (5–8). Indeed, in T1D islets, α cell function is compromised, whereas the few remaining β cells are functionally nearly normal (9).

Glucagon is the main secretory product of pancreatic α cells. The function of this peptide hormone is to increase glucose production and thus provide a sustained glucose supply to the brain and other vital organs during fasting conditions. In T1D, glucagon secretion is abnormal — sometimes abnormally elevated (10, 11). Thus, targeting of the pancreatic α cell and its main secretory product glucagon has potential as a treatment for diabetes (6). Glucagon secretion from α cells is affected by paracrine modulators such as intra-islet insulin (12), zinc (13), somatostatin secreted from δ cells (14), cAMP (15) and cAMP-activated ion channels (16), Ca^2+^ (17), cytokine effects (18), gap junctions (19), direct effects of glucose (14, 20), and GABA (21). Both β and α cells express ATP-sensitive K^+^ channels (K_ATP_) and together with the glucose-sensing enzyme glucokinase play an essential role in the regulation of insulin and glucagon secretion. Glucokinase activity in α cells is similar to that in β cells and has been attributed a role as a metabolic glucose sensor (22). Metabolism is nevertheless essential for glucose recognition, since glucose-inhibited glucagon secretion is mimicked by a glucokinase activator and secretion is not affected by a nonmetabolizable glucose transport analog (23). In support of this, a recent studies strongly indicates a direct role for glucose sensing via glucokinase in inhibition of glucagon secretion (20, 24, 25). In addition, glucagon secreted from α cells acts in a paracrine fashion on β cells, and this crosstalk is important for highly efficient glucose stimulation of insulin secretion (26–28). This said, our understanding of the progression of β and α cell dysfunction during the development of T1D is incomplete, due in part to the difficulty in obtaining T1D pancreata and islets for functional analysis.

**Figure 1:**
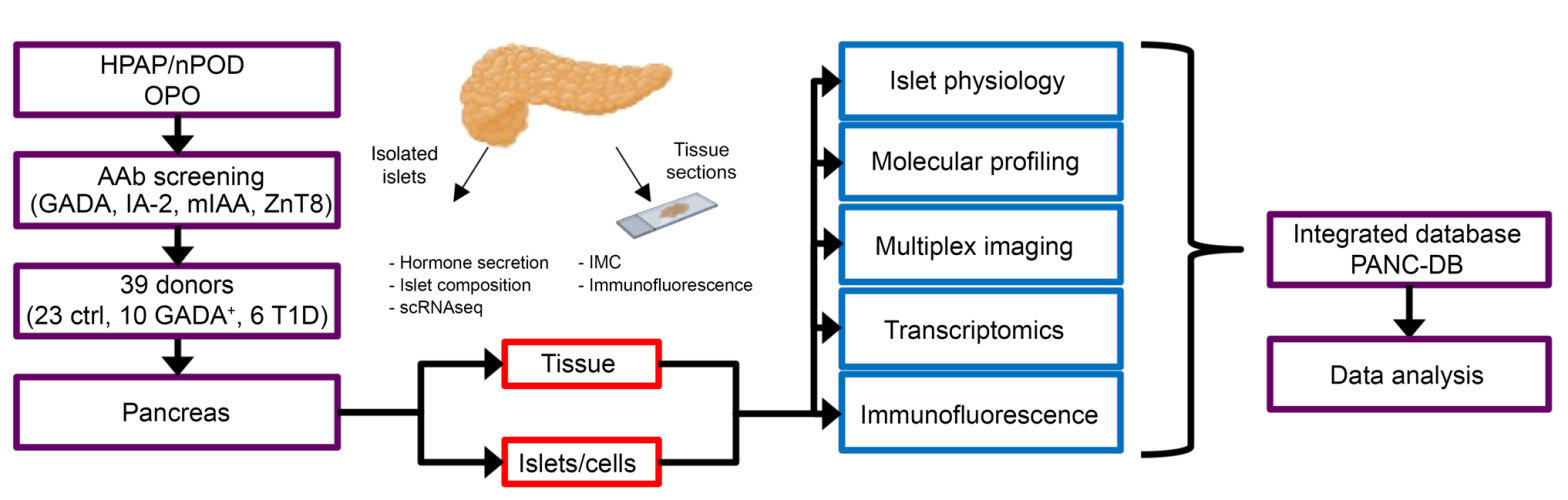
Study design and workflow. HPAP, working with Network for Pancreatic Organ Donors with Diabetes (nPOD), identifies organ donors of interest (recent-onset T1D, antibody^+^ donors, and control participants). For organ donors younger than 30 years of age without diabetes, the organ procurement organization (OPO), using HPAP/nPOD protocols and reagents, screens for the presence of AAbs (GADA, IA-2, mIAA, ZnT8). If a suitable organ donor is identified, pancreatic and immune tissues are shipped to Penn for processing. The tissue and islets are then analyzed for hormone secretion, multiplex imaging, molecular phenotyping, transcriptomics, and immunofluorescence staining at Vanderbilt and UPenn. All data are coregistered and integrated into a publicly accessible database (PANC-DB; https://hpap.pmacs.upenn.edu).

The Human Pancreas Analysis Program (HPAP; https://hpap.pmacs.upenn.edu), funded by the National Institute of Diabetes and Digestive and Kidney Diseases (NIDDK), NIH, provides comprehensive physiological and molecular profiling of the pancreas during T1D pathogenesis and publicly disseminates simultaneous analyses of human islet physiology, metabolism, molecular profiling, and immunobiology from well-characterized deceased donors (ref. 29 and Figure 1). Utilizing donor tissues and cells from the HPAP, we examined both insulin and glucagon secretion from nondiabetic, AAb^+^, and T1D islets. We made the striking observation of a distinct early defect in α cell function that precedes β cell loss during the progression of T1D, a finding that suggests that not only overt disease, but also the progression to T1D itself, is bihormonal in nature.

## Results

### Glucose sensitivity is preserved in the remaining β cells but lost in α cells of islets from T1D donors.

**Figure 2:**
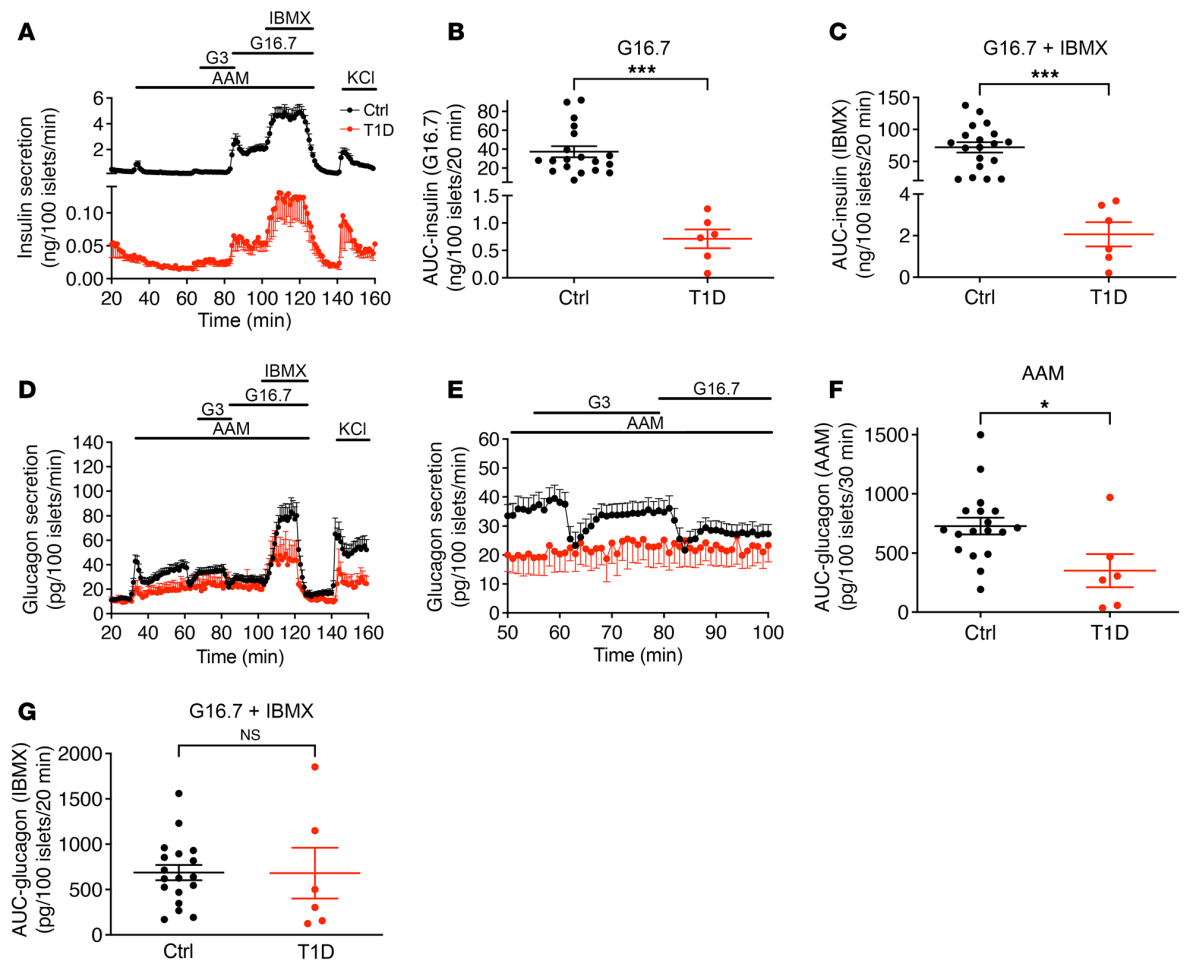
Insulin and glucagon secretion in islets from healthy and T1D donors. (**A**) The dynamics of insulin secretion in response to different stimuli. (**B**) Total insulin secretion during 16 mM glucose stimulation. (**C**) Total insulin secretion during IBMX potentiation. The basal and maximal insulin secretion in T1D islets was 1/60 that of normal islets. Notably, T1D islets had similar insulin response patterns at low glucose, high glucose, and with IBMX treatment compared with control islets. (**D**) Glucagon secretion profiles. (**E**) Magnified view of a selected section (53–100 min) of the experiment presented in **D** to highlight the difference in glucose suppression of glucagon secretion between normal and T1D islets. (**F**) Total glucagon secretion during AAM stimulation. (**G**) Total glucagon secretion during IBMX potentiation. *P < 0.05 and ***P < 0.001. Ctrl, control.

We performed insulin and glucagon secretion studies by perifusion of islets isolated from 19 normoglycemic organ donors with no AAbs against islet antigens (control), 9 normoglycemic organ donors positive for AAbs against GAD (GADA^+^), and 6 organ donors with T1D (Supplemental Table 1; supplemental material available online with this article; https://doi.org/10.1172/JCI156243DS1). Both basal and maximal rates of insulin secretion were dramatically reduced in T1D islets (1/60 that of normal islets) (Figure 2A). Remarkably, however, all stimuli (amino acids, low glucose, high glucose, and 3-isobutyl-1-methylxanthine [IBMX]) enhanced insulin secretion by T1D islets in a pattern similar to that seen in normal islets (Figure 2A; shown as a percentage of content in Supplemental Figure 1C). Total insulin secretion under high glucose stimulation was 0.95% that of control islets (Figure 2B), and IBMX potentiation was 1.47% that of control islets (Figure 2C). As expected, insulin content was markedly reduced in T1D islets (Supplemental Figure 1A).

Both the first and second phases of amino acid–stimulated glucagon secretion were significantly reduced in T1D islets (Figure 2, D and F; shown as a percentage of content in Supplemental Figure 1D). Neither low nor high glucose levels resulted in suppression of glucagon secretion (Figure 2E); however, there was no difference between T1D and control islets in terms of IBMX-potentiated glucagon secretion (Figure 2G), but depolarization by KCl caused a reduction in glucagon secretion in T1D islets.

### Glucose suppression of glucagon secretion is impaired in islets from GADA^+^ donors.

**Figure 3:**
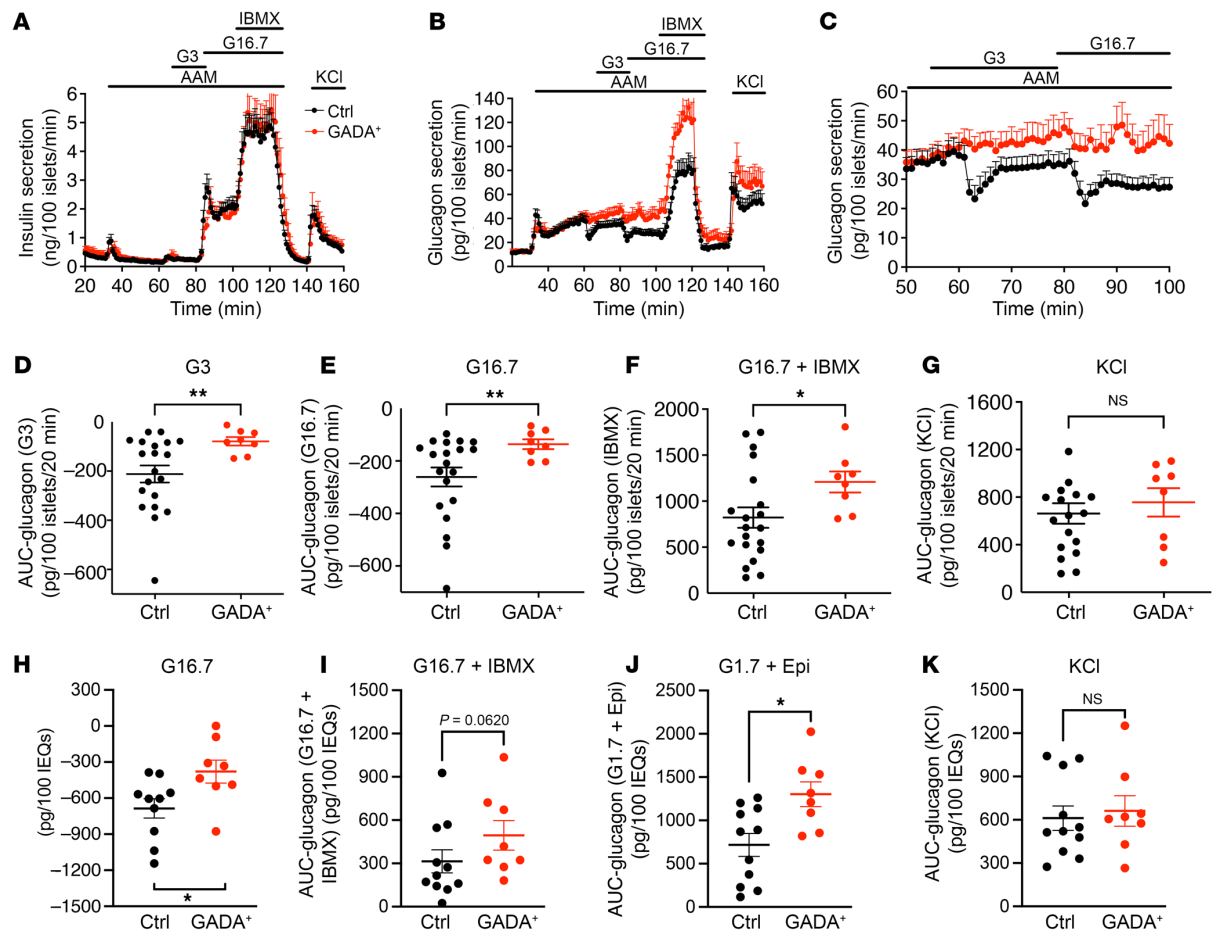
Insulin and glucagon secretion in islets from healthy and GADA^+^ donors. (**A**) The dynamics of insulin secretion during different interventions. (**B**) The dynamics of glucagon secretion. (**C**) Magnified view of a selected section (53–100 min) of the data from **B** highlights the difference in glucose suppression of glucagon secretion between normal and GADA^+^ islets. (**D**–**G**) Total glucagon secretion during 3 mM glucose (**D**), 16.7 mM glucose (**E**), G16.7 plus IBMX treatment (**F**), and KCl treatment (**G**) calculated as the AUC. (**H**–**K**) Islets from the same preparations were assessed by perifusion assay at Vanderbilt (see Supplemental Figure 2, A and B). AUC analysis of glucagon responses to high glucose (**H**), c-AMP–mediated secretion in response to IBMX (**I**), and epinephrine (**J**), and an unaltered KCl response (**K**). *P < 0.05 and **P < 0.01, by unpaired two-tailed *t* test. EQs, islet equivalents.

Next, we used 9 HPAP islet preparations from single GADA^+^, normoglycemic individuals to investigate potential early alterations in islet function. Islets from the same preparations were analyzed simultaneously at the University of Pennsylvania (hereafter referred to as Penn) and at Vanderbilt University (hereafter referred to as Vanderbilt) using 2 complementary perifusion protocols to maximize the information that could be obtained and to cross-validate our findings. The protocol developed by Brissova and colleagues was previously applied for functional phenotyping of T1D islets (9) and has been adopted by the Human Islet Phenotyping Program for functional analysis of human islet preparations made available for research by the Integrated Islet Distribution Program (IDPP) (https://iidp.coh.org/). The second protocol used at Penn (see Methods) was specifically designed to be sensitive to changes in the α cell response to amino acids and low glucose. At both laboratories, stimulated insulin secretion profiles were similar between the GADA^+^ and control cases (Figure 3A and Supplemental Figure 2, A and E–H; shown as a percentage of content in Supplemental Figure 1E).

In striking contrast to insulin secretion, glucagon secretion was substantially altered in GADA^+^ donors. Amino acids induced biphasic glucagon secretion to the same extent in control and GADA^+^ islets, with the second phase of glucagon secretion monotonically increasing during amino acid stimulation in both groups of islets (Figure 3B; shown as a percentage of content in Supplemental Figure 1F). Low and high glucose levels caused sustained suppression of glucagon secretion in control islets, as expected. Surprisingly, however, in GADA^+^ donor islets, low glucose levels effected only a slight delay in the monotonically increasing second phase of glucagon secretion, whereas high glucose levels caused little to no suppression of glucagon secretion (Figure 3C). AUC quantification showed that the suppression of glucagon secretion during low and high glucose levels was significantly lower in the GADA^+^ donors (Figure 3, D, E, and H). In addition, potentiation of glucagon secretion by IBMX, a phosphodiesterase inhibitor that maximally increases intracellular cAMP concentrations, was approximately 50% greater in islets from GADA^+^ donors than in control islets (Figure 3F). We observed no difference in the readily releasable pool of glucagon granules revealed by KCl stimulation (Figure 3G). Analysis of the same islet preparations using the Vanderbilt protocol confirmed a decreased suppression of glucagon secretion by glucose, an increased response to low glucose plus epinephrine, and a trend toward an increased response to IBMX, with no change in the KCl effect (Figure 3, H–K, and Supplemental Figure 2B). We also measured the content of insulin and glucagon in all islet preparations and found that the content of both insulin and glucagon was similar between control and GADA^+^ donor islets (Supplemental Figure 1, A and B).

### Islet composition is not altered in GADA^+^ donors.

**Figure 4:**
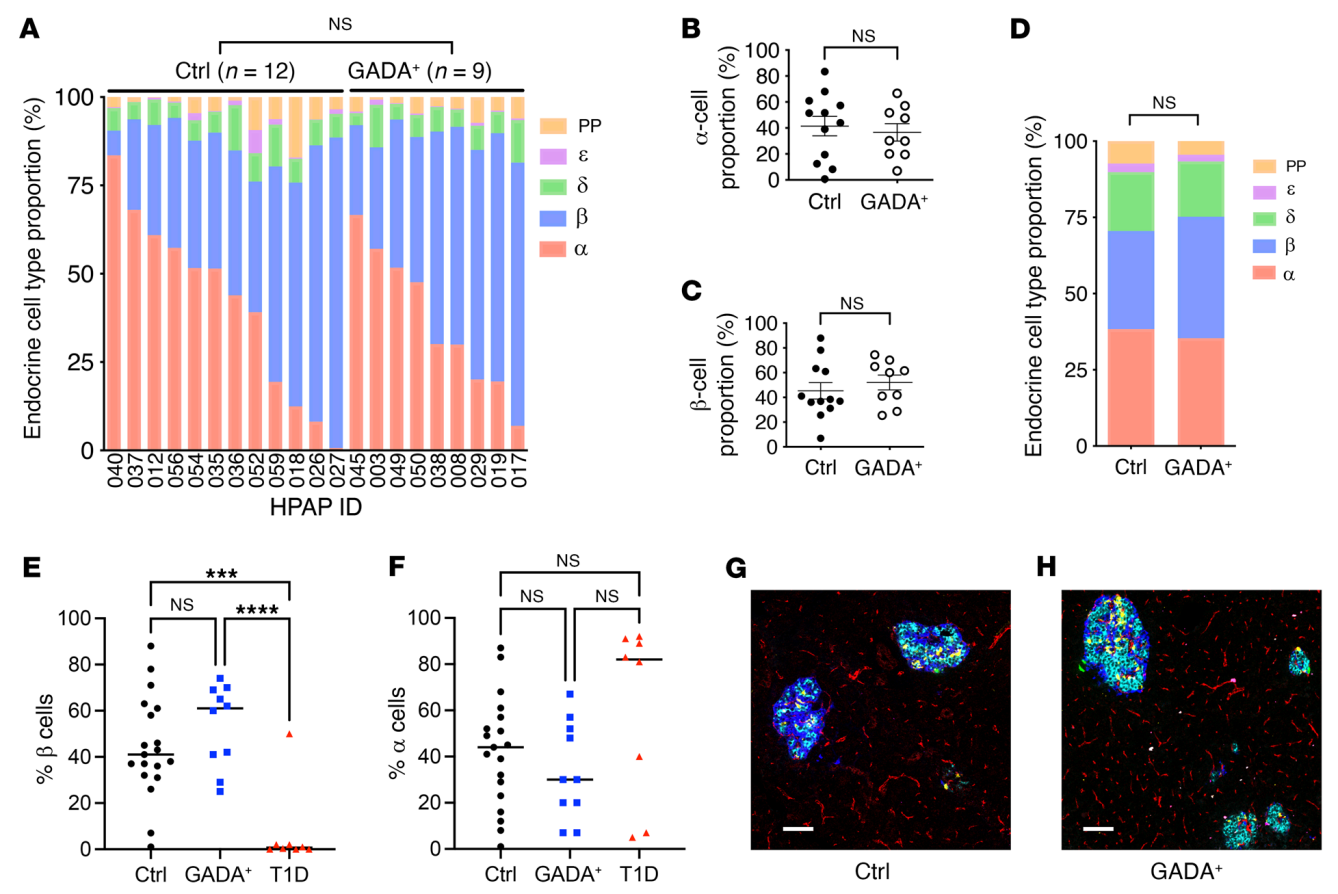
Composition of control and GADA^+^ donor islets. (**A**–**C**) Endocrine cell type proportions were determined by flow CyTOF of single-cell suspensions of islets. (**A**) Cell type fraction plotted for the indicated HPAP cases. (**B** and **C**) Average α (**B**) and β (**C**) cell percentage as determined by flow CyTOF. (**D**) Endocrine cell type proportions determined by IMC. (**E**) β Cell percentage of endocrine cells determined from IMC for islets from controls, GADA^+^, and T1D individuals. (**F**) α Cell percentage of endocrine cells determined from IMC for islets from controls, GADA^+^, and T1D individuals. ***P < 0.001 and ****P < 0.0001, by unpaired two-tailed *t* test. (**G** and **F**) Representative examples of IMC images of control (**G**) and GADA^+^ (**H**) pancreas with 6 channels shown (cyan, insulin-peptide; blue, glucagon; yellow, somatostatin; red, PECAM; magenta, pancreatic polypeptide; green, ghrelin). Scale bars: 100 μm.

A difference in islet composition could explain the altered glucagon secretion in islets from GADA^+^ organ donors. To evaluate this possibility, we took advantage of the highly quantitative endocrine cell composition data available through HPAP. First, we determined the proportions of endocrine cells in donor pancreata using flow cytometry by time of flight (CyTOF) of single islet cell suspensions stained simultaneously with a panel of 36 antibodies. In contrast to our previous observation of an increased α cell fraction in T1D donors compared with controls (30), flow CyTOF analysis of islets from 9 nondiabetic GADA^+^ and 12 control individuals revealed no statistically significant change in the α cell fraction in the GADA^+^ islets (Figure 4, A–C, and Supplemental Figure 1, C and D). Second, we analyzed data from imaging mass cytometry (IMC) performed on tissue slides from the same donor; these images were segmented into thousands of individual cells, which were then identified by their protein marker expression. Again, as shown in Figure 4, D–F, and Supplemental Figure 3, A and B, there was no statistically significant difference in endocrine cell populations between the 2 groups. Figure 4, G and H, shows representative multichannel overlays of pancreatic islets from control and GADA^+^ organ donors that illustrate this point. To ascertain whether immunological infiltrates in or near islets are already present at the single GADA^+^ stage, we quantified immune cell infiltrates from our IMC images and performed detailed image analysis of the distance relationships between pancreatic islets and CD4^+^ and CD8^+^ T cells, proliferating (activated) and nonproliferating macrophages, B cells, and Tregs but found no significant differences (Supplemental Figure 4).

### Transcriptome alterations in α cells from GADA^+^ donors.

**Figure 5:**
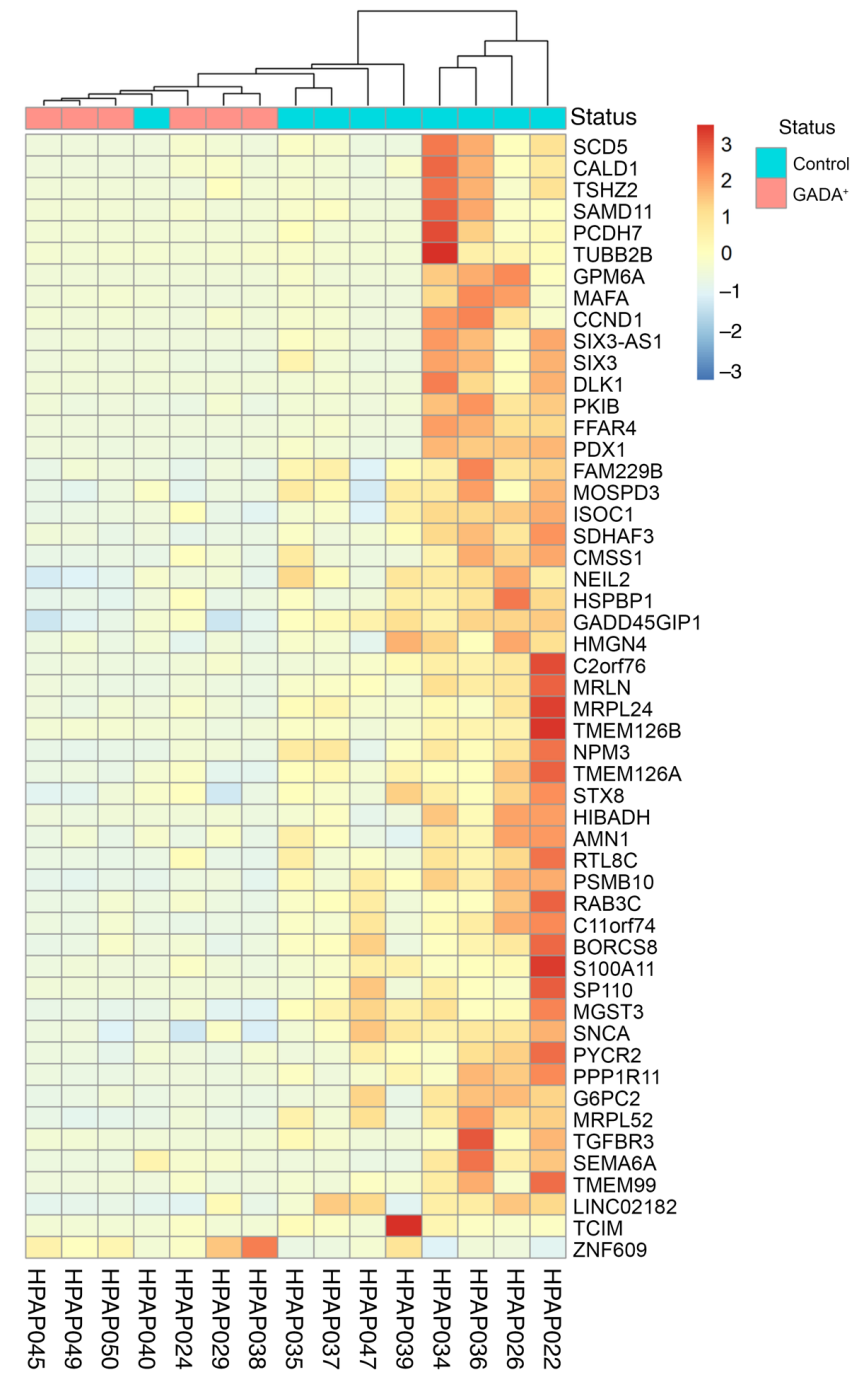
Single-cell transcriptome analysis and immunofluorescence staining of control and GADA^+^ α cells. Heatmap of hierarchical clustering of differentially expressed genes obtained by comparing pseudobulk α cell gene expression of 9 control and 6 GADA^+^ organ donors. This heatmap was generated with the R package heatmap.

Given that islet endocrine cell composition, islet architecture, and immune cell infiltration were not significantly altered in GADA^+^ donors, we next focused on α cell transcriptome changes to determine if they explain the observed α cell dysfunction. We analyzed single-cell RNA-Seq (scRNA-Seq) data from control and GADA^+^ organ donors produced by HPAP, and after cell type clustering, we selected only cells in the major α cell cluster (data not shown). Next, we collapsed the transcriptome of all single α cells for each donor into “pseudobulk” α cell transcriptomes and performed differential gene expression analysis. Overall, we found that the α cell transcriptomes for the 2 groups were quite similar; however, specific genes and pathways were differentially expressed, as shown in Figure 5. The heatmap in Figure 5 shows 52 differentially expressed genes identified by DESeq2. Most notable among the differentially expressed genes is cAMP-dependent protein kinase inhibitor β (*PKIB*), encoding the cAMP-dependent protein kinase inhibitor β, which acts as a competitive inhibitor of protein kinase A (Figure 5 and Supplemental Table 2). *PKIB* transcripts were decreased on average 4.9-fold in α cells from GADA^+^ organ donors, suggesting a possible activation of the cAMP pathway, consistent with the IBMX and epinephrine effect on glucagon secretion reported above.

**Figure 6:**
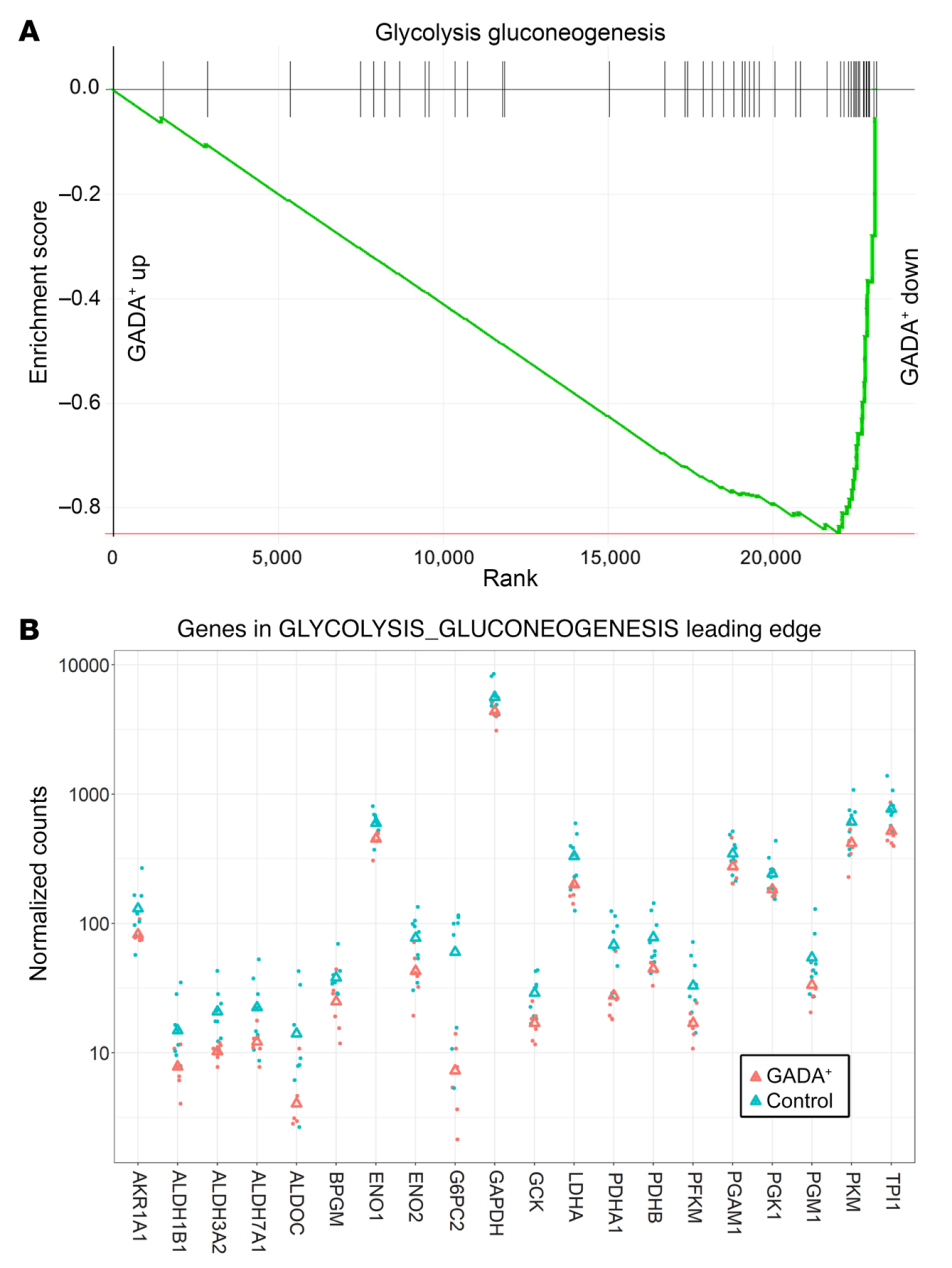
GSEA of glycolysis and gluconeogenesis genes in GADA^+^ donors compared with controls. (**A**) Genes annotated to the “glycolysis_gluconeogenesis” pathway are highly enriched among the genes downregulated in α cells from GADA^+^ organ donors. up, upregulated; down, downregulated. (**B**) Expression of the top 15 genes in the leading edge of the “glycolysis_gluconeogensis” pathway. Triangles indicate the mean.

**Figure 7:**
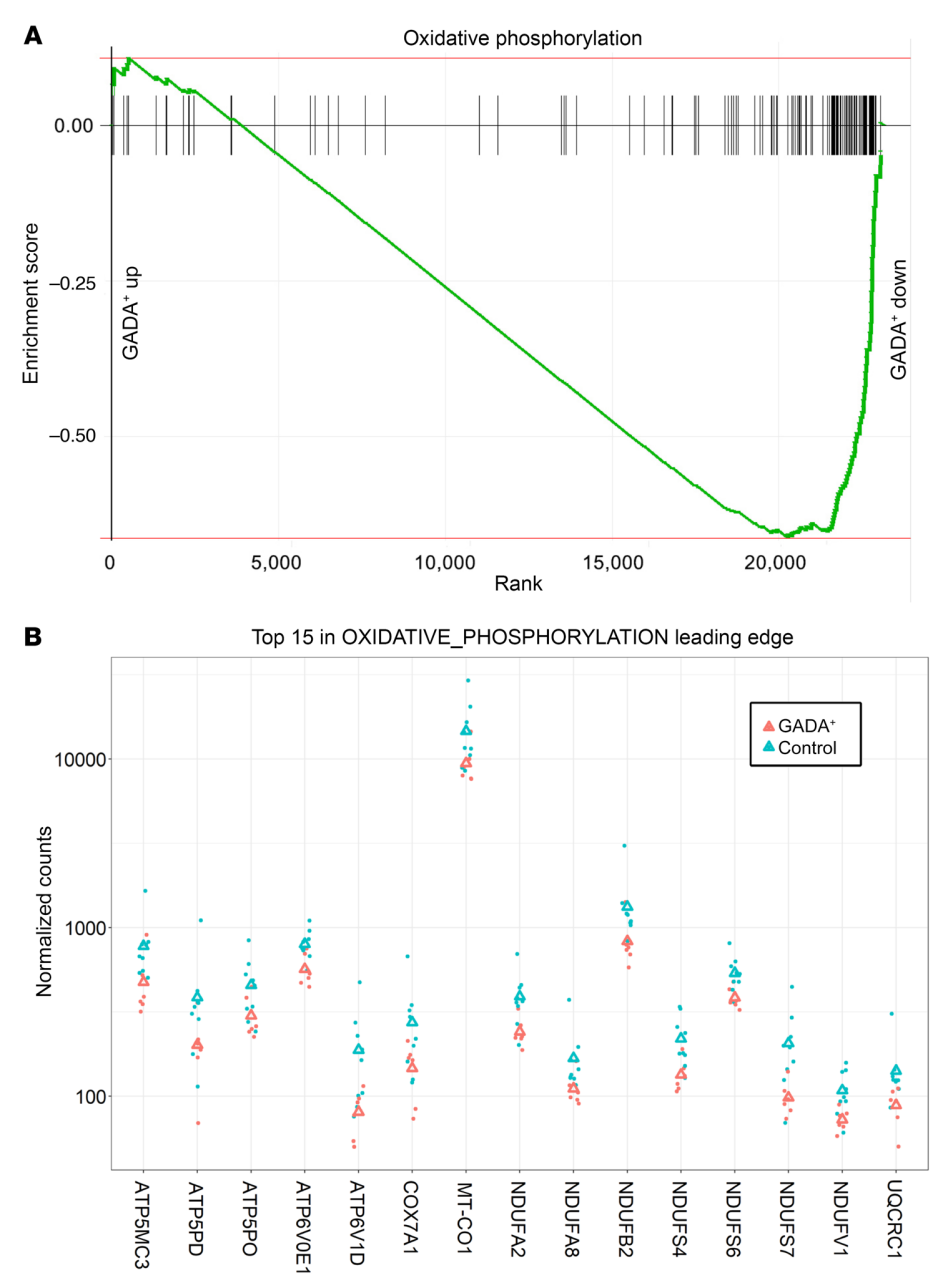
GSEA of oxidative phosphorylation genes in GADA^+^ donors compared with controls. (**A**) Genes annotated to the oxidative phosphorylation pathway were highly enriched among the genes downregulated in α cells from GADA^+^ organ donors. (**B**) Expression of the genes in the leading edge of the “oxidative_phosphorylation” pathway. Triangles indicate the mean.

Next, we used gene set enrichment analysis (GSEA), a method that detects statistically significant changes in transcript levels for preselected groups of functionally linked genes. The “glycolysis_gluconeogenesis” pathway was enriched among genes with lower transcript levels in α cells from GADA^+^ organ donors (Figure 6A; adjusted *P* = 0.0002). The genes in the leading edge of this pathway are shown in Figure 6B. Remarkably, the entire set of glycolysis genes, from *GCK* to *PDH*, was downregulated in α cells from GADA^+^ donors. It was recently demonstrated that the rate of glycolytic flux via glucokinase and thus ATP production determines the set point for inhibition of glucagon secretion by glucose (20, 24, 25). Oxidative phosphorylation was one of the most significantly affected pathways; genes in this pathway were downregulated in α cells from GADA^+^ organ donors (Figure 7A; adjusted *P* = 0.008). The leading edge of this pathway (i.e., the core group of genes that accounts for the gene set’s enrichment signal) contains 75 genes, the top 15 of which are shown in Figure 7B. Among these genes are 3 that encode subunits of the mitochondrial ATP synthase as well as 7 that encode subunits of the NADH:ubiquinone oxidoreductase complex (complex 1), which may indicate a reduction in mitochondrial oxidative ATP synthesis.

**Figure 8:**
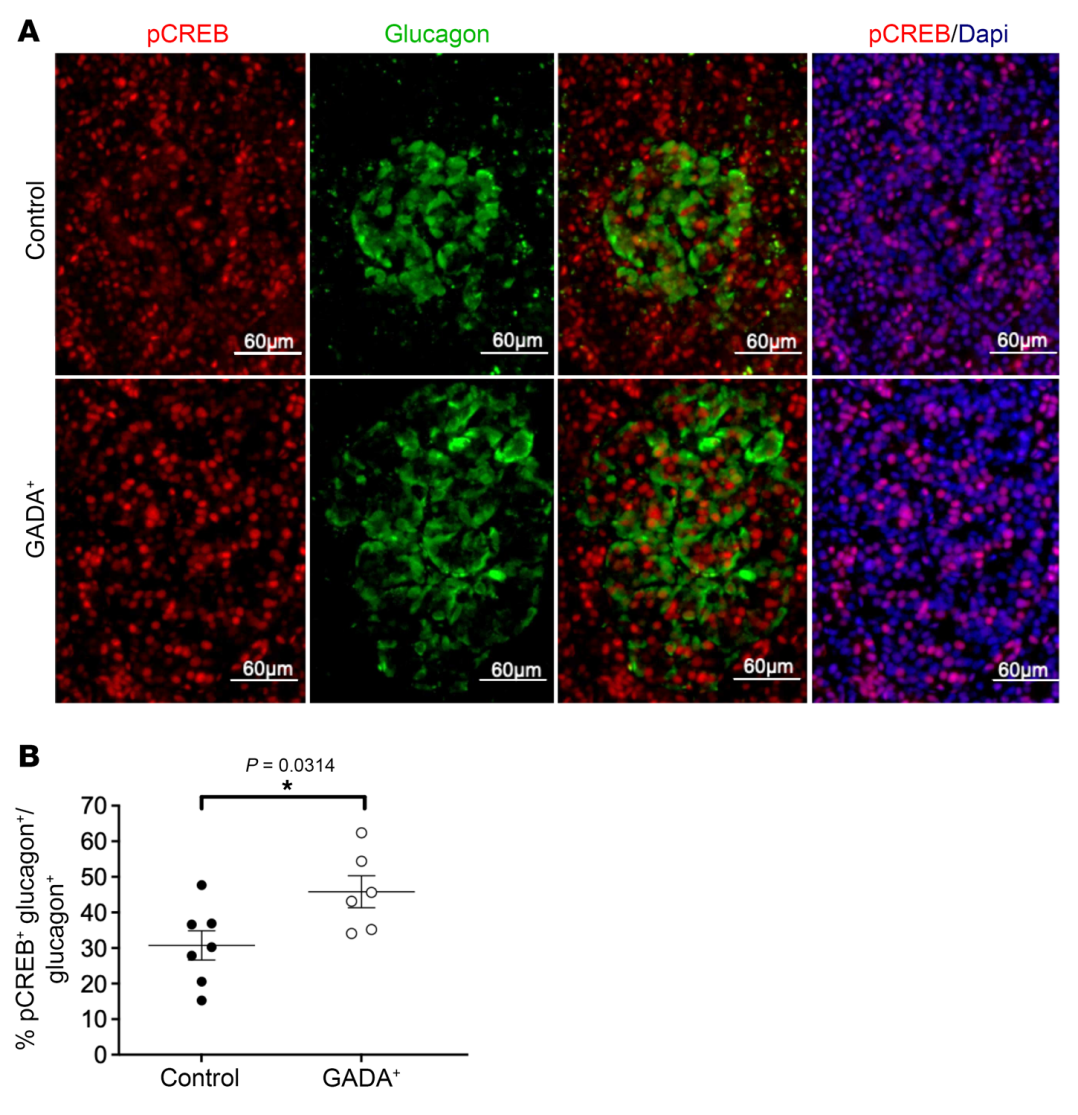
p-CREB staining in pancreatic sections from control and GADA^+^ donors. (**A**) Immunofluorescence staining of pancreatic sections with antisera against p-CREB and glucagon. Scale bars: 60 μm. (**B**) Nuclear p-CREB^+^ α cells were increased in frequency in GADA^+^ islets (*n* = 7 for control islets and *n* = 6 for GADA^+^ islets). *P < 0.05, by unpaired two-tailed *t* test.

The increased potentiation of glucagon secretion by IBMX and epinephrine described above (Figure 3, B, F, I, and J, and Supplemental Figure 2B) suggested an upregulation of the cAMP signaling pathway in α cells from GADA^+^ donors. As introduced above, we identified *PKIB*, a competitive inhibitor of protein kinase A, as the most differentially expressed in the α cells of GADA^+^ islets, providing a possible molecular explanation for this effect. To provide further evidence for altered cAMP signaling in α cells of GADA^+^ individuals, we performed immunofluorescence staining of human islet sections using anti–phosphorylated c-AMP response element–binding protein (anti–p-CREB) antisera. Elevated cAMP stimulates the activity of protein kinase A, which in turn phosphorylates the transcription factor CREB. The percentage of α cells that expressed nuclear p-CREB was significantly elevated in GADA^+^ islets (Figure 8).

## Discussion

T1D donor islets showed preserved stimulation of insulin secretion but dysfunction of α cells in T1D donors (9). Our data indicate that functional defects in the suppression of glucagon secretion are seen even earlier in T1D pathogenesis in nondiabetic, normoglycemic GADA^+^ donors. This GADA^+^ donor α cell phenotype is accompanied by altered α cell gene expression, particularly in glycolysis and oxidative phosphorylation, and altered cAMP signaling. Altered cAMP signaling was evidenced by an elevated IBMX response and by an increased percentage of p-CREB–expressing α cells. This study exemplifies the unique opportunity provided by the HPAP program to analyze nondiabetic GADA^+^ donors.

Whereas the metabolic regulation of insulin secretion is well understood (31), the mechanisms underlying the control of glucagon secretion by glucose are not well elucidated. Our gene expression analysis shows that both glycolysis and oxidative phosphorylation genes were significantly downregulated (*P* = 0.0002 and 0.0084, respectively), predicting less efficient glucose metabolism and ATP synthesis. It has been shown that α cells contain many K_ATP_ channels and already have a high ATP/ADP ratio in the absence of glucose (32, 33). This K_ATP_ channel activity within a “narrow window” is critical for the regulation of glucagon secretion (34). At low glucose concentrations, a subpopulation of the K_ATP_ channels present on the α-cell plasma membrane is open and sets the membrane potential to −60 mV, causing successive activation of the T-type Ca^2+^ channels, tetrodotoxin-sensitive (TTX-sensitive) Na^+^ channels, and L- and N-type Ca^2+^ channels (35, 36), resulting in glucagon secretion. By contrast, at high glucose levels, the rise in the intracellular ATP/ADP ratio results in closure of the K_ATP_ channels (37), which depolarizes the α cell membrane potential beyond the narrow window, causing voltage inactivation of previously activated ion channels involved in the depolarization cascade and leading to suppression of glucagon secretion. Thus, the regulation of K_ATP_ channels by ATP plays a major role in the regulation of glucagon secretion at both low and high glucose levels.

The decreased expression of glucokinase in GADA^+^ donor α cells may indicate a defect in glucose sensing. It was recently demonstrated that the rate of glycolytic flux via glucokinase and thus ATP production determines the set point for inhibition of glucagon secretion by glucose (20, 24, 25). Therefore, if GADA^+^ α cells produce ATP from glucose less efficiently because of lower glycolytic flux and oxidative phosphorylation, then a given concentration of glucose would less efficiently suppress glucagon secretion, providing a possible mechanism for the functional defect.

In addition to glycolysis and oxidative phosphorylation, cAMP signaling was also altered in GADA^+^ donor islets. Although islets from GADA^+^ donors had a normal insulin secretory profile, cAMP-mediated glucagon secretion was increased in response to both the phosphodiesterase inhibitor IBMX and to adrenergic stimulation with epinephrine. Consistent with these findings, transcripts encoding the potent protein kinase A (PKA) inhibitor *PKIB* were decreased in α cells from GADA^+^ donors, and GADA^+^ islets had an increased proportion of α cells expressing p-CREB. Glucose suppression of glucagon secretion can be overcome by maintaining cAMP at high levels (15). Thus, the elevation of cAMP pathway activity — as indicated by IBMX and epinephrine effects, gene expression analysis, and p-CREB staining — could also have contributed to the loss of glucose suppression of glucagon secretion in GADA^+^ donor islets. Further studies are needed to establish the causal link between elevated cAMP activity and dysfunction in glucagon secretion, including direct measurement of cAMP levels in α cells and/or modulation of PKA activity.

The literature suggests other possible mechanisms for glucose suppression of glucagon secretion (12–14, 16–19, 21). Among these, activation of GABA shuttle, which is present in human islets (38) and whose metabolic pathway includes GAD, results in membrane hyperpolarization and suppression of glucagon secretion (12, 39). Nevertheless, the regulation of glucagon secretion is multifactorial (40) and yet to be fully understood. Despite a lack of consensus on the mechanism, glucagon is known to be one of the key hormones in the pathophysiology of diabetes (5), and its role throughout the development of T1D should be further studied.

It is perhaps worth noting that our perifusion protocol was developed to better reveal differences in glucagon suppression by glucose. We used a physiological mixture of amino acids in order to depolarize the α cell (41), resulting in the stimulation of glucagon secretion from α cells. Subsequent addition of low and then high glucose concentrations to the physiological mixture of amino acids then leads to the suppression of glucagon secretion via increased glycolytic flux.

Historically, the development of diabetes has been characterized by defects in β cells. Through our phenotyping of nondiabetic, GADA^+^ islets, we discovered that α cell dysfunction preceded β cell loss. This study highlights the need to consider T1D as a disease in which both β and α cells are affected. Future studies will be needed to expand on the mechanisms suggested above through the lenses of α cell physiology, immune signaling, and paracrine effects. In addition, given that only a subset of individuals positive for single AAbs progress to develop T1D, further investigation involving a larger number of AAb^+^ individuals is needed to define the possible distinctions between progressors and nonprogressors. This study pertains to adult GADA^+^ individuals, and it would be important to study children with GADA positivity. With a comprehensive understanding of the role of the α cell in diabetes, a more targeted intervention may be developed for the critical single AAb^+^ stage.

## Methods

### Human islets.

GADA^+^ organ donors were screened by the Network for Pancreatic Organ Donors with Diabetes (nPOD)/HPAP team, as previously described (42). Human islets were isolated from donor pancreata using standard multiorgan recovery and modified Ricordi techniques (43) at the accredited Human Islet Resource Center at Penn. Following 2 to 3 days of culturing in supplemented CMRL-1066 medium (44, 45), we characterized the physiology and metabolic state of 39 human islet preparations isolated from 23 controls (*n* = 20 from HPAP, *n* = 3 from Penn’s Human Islet Resource Center; age, 27 ± 10 years; BMI, 27 ± 7 kg/m^2^; hemoglobin A1c [HbA1c], 5.3 ± 0.6; C-peptide, 7.9 ± 5.7 ng/mL [mean SD]; *n* = 19 Whites, *n* = 3 Blacks, *n* = 1 Hispanic individual, *n* = 14 males, *n* = 9 females); 10 GADA^+^ individuals (age, 24 ± 5 years; BMI, 27 ± 4 kg/m^2^; HbA1c, 5.4 ± 0.2; C-peptide, 7.8 ± 7.2 ng/mL; *n* = 8 Whites, *n* = 1 Blacks, *n* = 1 Hispanic individual, *n* = 7 males, *n* = 3 females); and 6 T1D donors (age, 20 ± 8 years; BMI, 19 ± 5 kg/m^2^; HbA1c, 10.1 ± 0.6; C-peptide, 0.2 ± 0.2; *n* = 4 Whites, *n* = 2 Hispanic individuals, *n* = 4 males, *n* = 2 females). Only donors under the age of 40 years were included in this study. An aliquot of most islet preparations was also shipped to Vanderbilt for parallel analyses.

### Perifusion of human islets.

HPAP uses 2 complementary and validated islet perifusion protocols to assess insulin and glucagon secretion (at both Penn and Vanderbilt). At Penn, islets were preperifused with substrate-free medium. Next, a physiological amino acid mixture (AAM) consisting of 19 amino acids (46) at a total concentration of 4 mM was added to stimulate glucagon secretion. Then, low and high glucose concentrations (3 mM and 16.7 mM, respectively) were added to stimulate insulin secretion and to inhibit glucagon secretion. During the high glucose step, IBMX (0.1 mM) was added to maximally increase intracellular cAMP levels and stimulate the secretion of both hormones. Finally, a brief washout period with substrate-free medium removed all stimulants, and then 30 mM KCl was added to depolarize the islet cells and quantify the readily releasable pool of secretory granules. Insulin and glucagon in perifusion aliquots and insulin content were measured as previously described (47). Glucagon content was measured by ELISA (Crystal Chem).

At Vanderbilt, islet function was assessed by perifusion as previously described (9), and this methodology was adopted by the NIDDK-funded Human Islet Phenotyping Program (HIPP) of the IDPP (https://www.protocols.io/view/analysis-of-islet-function-in-dynamic-cell-perifus-bt9knr4w). Insulin and glucagon concentrations in perifusates and islet extracts were measured by radioimmunoassay (insulin, RI-13K; glucagon, GL-32K; MilliporeSigma).

### scRNA-Seq analysis.

The Single Cell 3′ Reagent Kit, version 2 or 3, was used to generate scRNA-Seq data. Three thousand cells per donor were targeted for recovery. All libraries were validated for quality and size distribution using a BioAnalyzer 2100 (Agilent Technologies) and quantified using the KAPA Library Quantification kit (Illumina). For samples prepared using the Single Cell 3 Reagent Kit, version 2, the following chemistry was performed on an Illumina HiSeq 4000: read 1: 26 cycles; i7 index: 8 cycles; i5 index: 0 cycles; and read 2: 98 cycles. For samples prepared using the Single Cell 3 Reagent Kit, version 3, the following chemistry was performed on an Illumina HiSeq 4000: read 1: 28 cycles; i7 index: 8 cycles; i5 index: 0 cycles; and read 2: 91 cycles. Cell Ranger (10x Genomics, version 3.0.1) was used for bcl2fastq conversion, alignment (using the hg38 reference genome), filtering, counting, cell calling, and aggregation (--normalize=none). scRNA-Seq data were preprocessed as described previously (48). The resulting Seurat (49, 50) object was downloaded from the cellxgene resource (https://cellxgene.cziscience.com/collections/51544e44-293b-4c2b-8c26-560678423380). Exclusion of donor HPAP-019 (whose scRNA-Seq library was from sorted β cells) and donors younger than 5 years of age or older than 40 years of age resulted in 9 healthy control donors and 6 GADA^+^ donors (Supplemental Table 1). Using R, version 4.1.1, pseudobulk raw counts were generated for each gene by aggregating (via sum) counts of the α cells at the donor level. Raw counts were then input into DESeq2 (51) for preprocessing and differential expression analysis, with a *P* value threshold of 0.05. GSEA (52, 53) was performed using the fgsea Bioconductor package (54), with gene ranking based on the shrunken log_2_ fold changes derived using the apeglm Bioconductor package (55). The Kyoto Encyclopedia of Genes and Genomes (KEGG) canonical pathway was used for collection (c2.cp.kegg.v7.4.symbols.gmt) from MSigDB (52), augmented with the cAMP signaling pathway directly extracted from the KEGG (56), for a total of 187 gene sets.

### Flow CyTOF.

The flow CyTOF experiments were performed as described previously (57). Briefly, after dissociated cells were barcoded according to the manufacturer’s protocol (Fluidigm, 101-0804 B1), they were labeled with 36 metal-conjugated antibodies in FoxP3 permeabilization buffer (eBioscience, 00-8333) with 1% FBS (Hyclone, catalog 7207) for 12 hours at 4°C at a concentration of up to 3 million cells per 300 μL antibody cocktail, followed by washing twice with FoxP3 permeabilization buffer. Cells were then incubated with the DNA intercalator iridium (Fluidigm, 201192A) at a dilution of 1:4000 in 2% paraformaldehyde (Electron Microscopy Sciences, 15714) in Dulbecco’s PBS (Corning, 21-031-CV) at room temperature for 1 hour. Mass cytometric data were acquired on a Fluidigm Hyperion instrument. Flow CyTOF data analyses of endocrine and immune cell composition were performed using the Cytobank platform (https://www.cytobank.org/).

### IMC.

IMC was performed as described previously (30, 58). Tissue slides were labeled with 31 metal-conjugated antibodies and then ablated by a UV laser, and the resulting plumes of particles were carried to a mass spectrometer for signal detection. Cell segmentation was performed with Vis image analysis software (Visiopharm). Noise was first removed from all images with a 3 × 3 pixel median filter, followed by nuclear object detection using a polynomial local linear parameter-based blob filter applied to the iridium-193 DNA channel of each region of interest (ROI). Nuclear objects of less than 10 μm^2^ were filtered out, and then the remaining objects were expanded to a maximum of 7 pixels before exporting the mean channel intensities for further analysis. Each ROI was individually *z* score normalized prior to cluster analysis, which was performed with Phenograph using the 5 hormone channels (C-peptide, ghrelin, glucagon, somatostatin, and pancreatic polypeptide [PP]) as input and a nearest neighbor setting (k) of 200. Endocrine cell types were assigned according to the expression of their canonical hormones and quantified.

### Immunofluorescence staining for p-CREB.

Paraffin-embedded, fixed pancreatic sections affixed to glass slides were dewaxed, and heat-induced epitope retrieval was performed using citrate buffer. Sections were blocked with 5% donkey serum in the presence of 0.1% Triton followed by overnight incubation with primary antisera at 4°C (p-CREB, Cell Signaling Technology; glucagon, Abcam). After extensive washing, sections were incubated with secondary antisera (Jackson ImmunoResearch) for 1 hour at room temperature. Imaging was performed using the inverted microscope on the BioTek Cytation 5 Cell Imaging Multimode Reader. Using the companion BioTek Gen5 software, background flattening followed by constant threshold correction were applied to all images. A total of 2636 and 3523 glucagon^+^ cells from control and GADA^+^ donors, respectively, were manually counted, and p-CREB expression within this group was evaluated. The data are expressed as a percentage of glucagon and p-CREB double-positive cells compared with total glucagon^+^ cells in control and GADA^+^ donors. An unpaired two-tailed *t* test was used to compare the groups.

### Statistics.

Insulin and glucagon data are presented as the mean ± SEM. The AUC for each intervention was calculated by case, and statistical analysis was then performed using an unpaired two-tailed *t* test with Welch’s correction. In appropriate cases, significant differences between groups were determined by ANOVA with post hoc analysis using Dunnett’s multiple-comparison test (59). A *P* value of 0.05 or less was considered significant. IGOR data analysis software (60) (Wavemetrics) was used to measure total insulin or glucagon secretion by calculating the integral under the insulin and glucagon curves, respectively (presented in relative units).

### Study approval.

The Penn IRB considered this research exempt, because the pancreatic tissues were received from deceased, deidentified organ donors. All pancreata were acquired from deceased donors after obtaining consent from their families through the United Network for Organ Sharing (UNOS) (61).

## Author contributions

NMD, FMM, MB, KHK, and DAS conceptualized the study. NMD, AVR, WQ, DT, and JL performed experiments. NMD, AVR, DT, JL, EM, and JR conducted imaging and visualization procedures. NMD, JR, DAS, and KHK were wrote the original draft of the manuscript. MAA, ACP, MB, KHK and DAS reviewed and edited the manuscript. JR, LG, EM, and GV conducted formal analyses. MLG was responsible for project administration. CL and AN provided critical resources. MB was responsible for validation of the data. AN, MAA, ACP, KHK, MB and DAS acquired funding for the study. MB, KHK and DAS supervised the study.

## Supplementary Material

Supplemental data

ICMJE disclosure forms

## Version 1 06/01/2022

Electronic publication
