# Supplementary material for: α Cell dysfunction in islets from nondiabetic, glutamic acid decarboxylase autoantibody–positive individuals
Source: J Clin Invest. 2022 Jun 1;132(11):e156243. doi: 10.1172/JCI156243 (PMC9151702; doi:10.1172/JCI156243)
Supplement: Supplemental data [file jci-132-156243-s040.pdf]

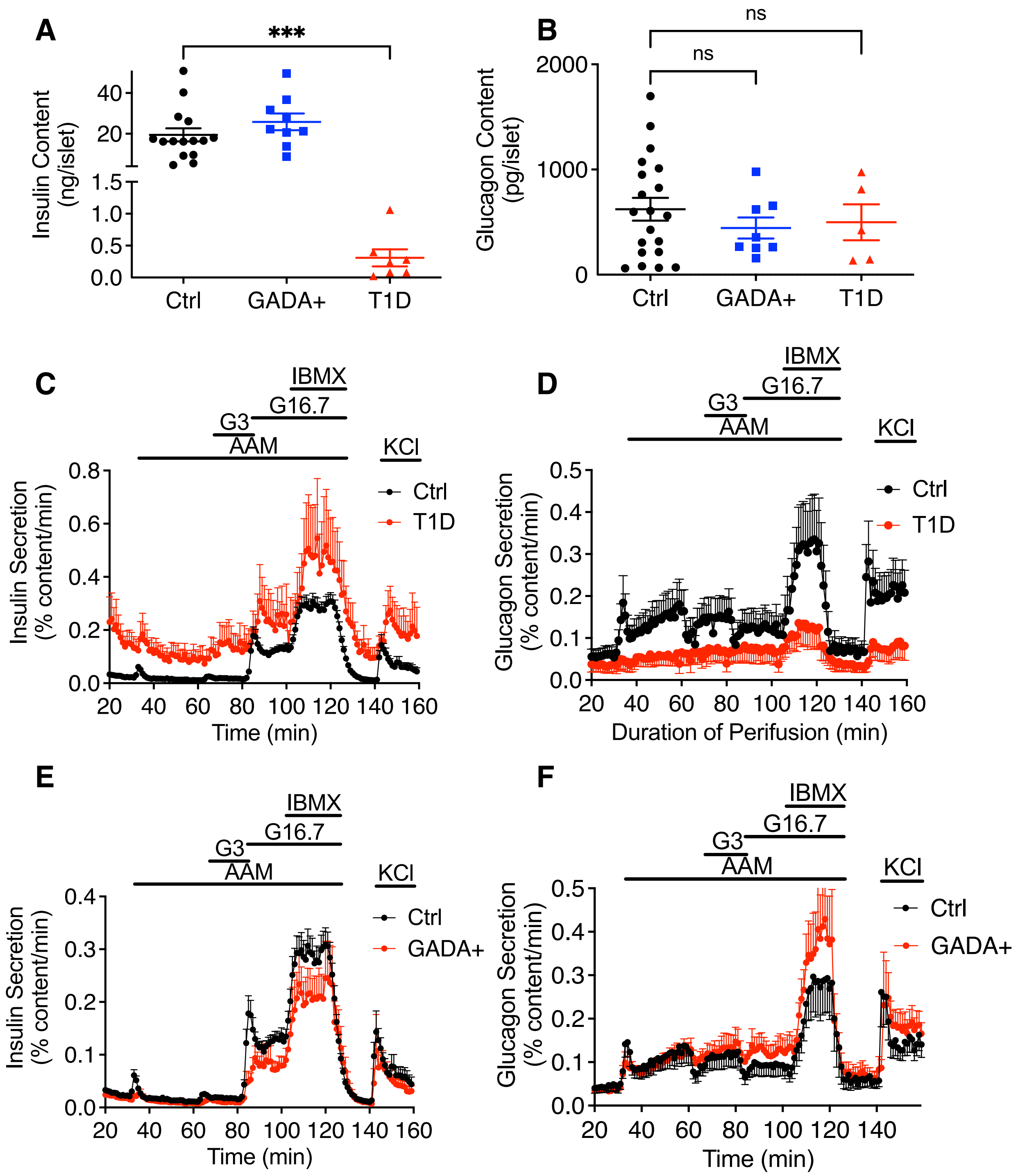

Supplemental Figure S1. (A) Insulin and (B) glucagon content of islets recovered after perifusion. (C) Insulin secretion profiles from perifusion of isolated islets of control and T1D donors, as percentage of total insulin content. (D) Glucagon secretion profiles from

perifusion of isolated islets of control and T1D donors, as percentage of total glucagon content. (E) Insulin secretion profiles from perifusion of isolated islets of control and GADA+ donors, as percentage of total insulin content. (F) Glucagon secretion profiles from perifusion of isolated islets of control and GADA+ donors, as percentage of total glucagon content.

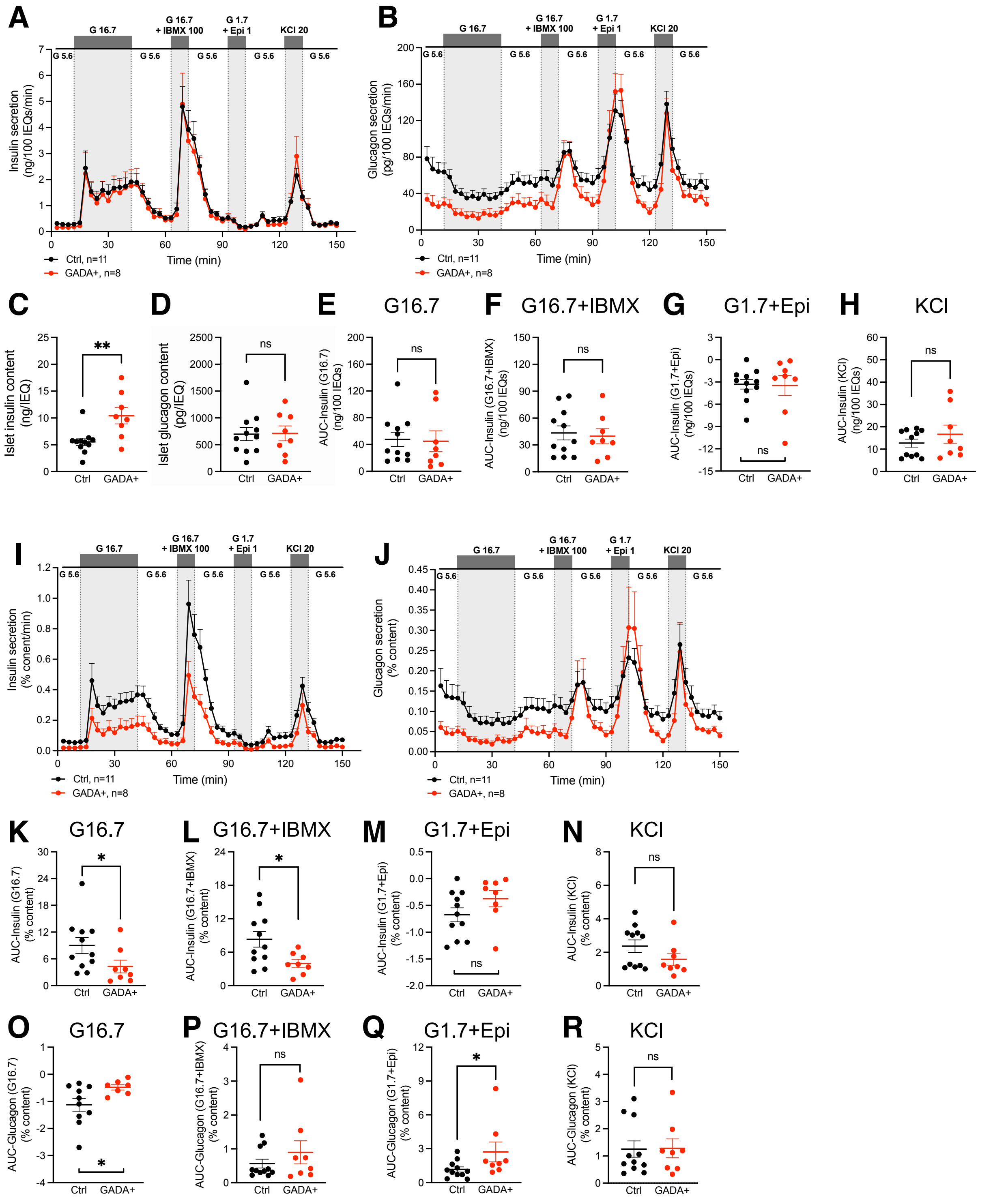

Supplemental Figure S2. Secretory profiles of islets from GADA+ organ donors and controls assessed at Vanderbilt University. Dynamic insulin (A) and glucagon (B) secretory response to various secretagogues measured by perifusion of control (Ctrl) and

GADA+ islets; G 5.6 – 5.6 mM glucose; G 16.7 – 16.7 mM glucose; G 16.7 + IBMX 100 – 16.7 mM glucose with 100 μM isobutylmethylxanthine (IBMX); G1.7 + Epi 1 – 1.7 mM glucose and 1μM epinephrine; KCl 20 – 20 mM potassium chloride (KCl) was normalized to islet volume expressed by islet equivalents (IEQs); 1 IEQ corresponds to an islet with a diameter of 150 μm. (C) Islet insulin content. (D) Islet glucagon content. (E-H) Integrated area under the curve (AUC) analyses to insulin secretagogues highlighted in gray in panel A. * $p<0.05$; ** $p<0.01$. Error bars indicate SEM. Panels C-H were analyzed by two-tailed t-test. (I) Insulin secretion profiles as percentage of total insulin content. (J) Glucagon secretion profiles as percentage of total glucagon content. (K-N) AUC analyses of insulin secretion highlighted in gray in panel I. (O-R) AUC analyses of glucagon secretion highlighted In gray in panel J.

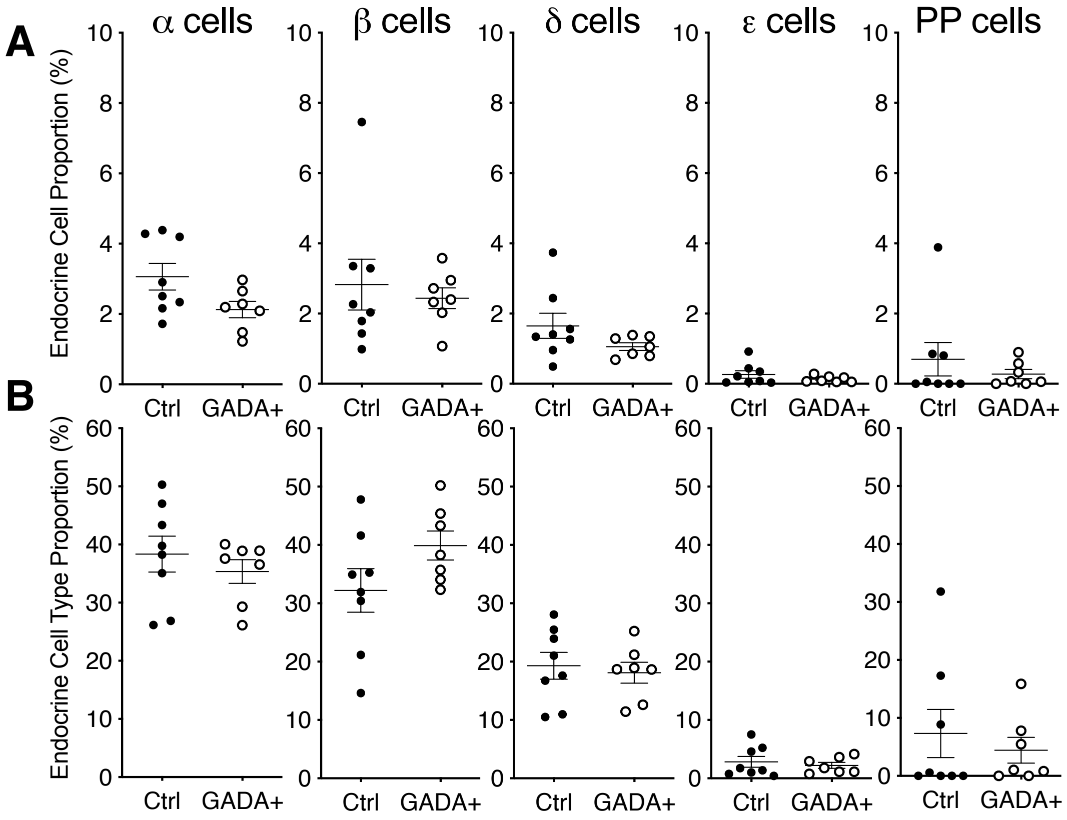

Supplemental Figure S3. Quantification of cell type proportions for all donors, determined by IMC. Panels represent proportions of each endocrine cell type as a proportion of (A) total cell number and (B) the endocrine cells.

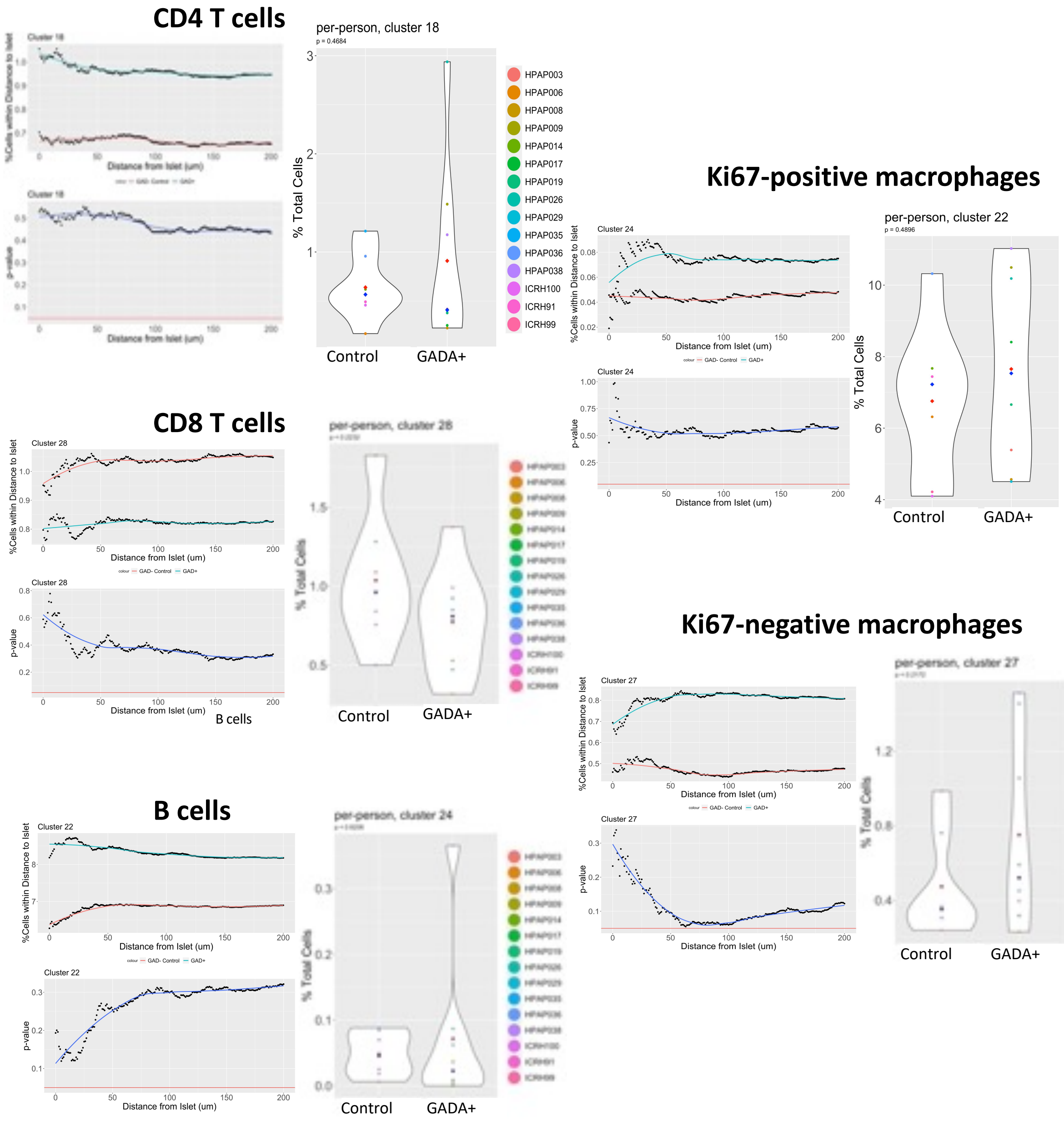

Supplemental Figure S4. Immune cell infiltration as determined by imaging mass cytometry (IMC). For various immune cell types, cell distributions by distance-from-islet were determined. Violin plots of mean distance by cell type and donor are also presented. P-values for comparison between GADA+ donors and controls are shown in the lower-left of each panel.

Supplemental Table S1. Donor Information.

ID	Recovery Center	Sex	Age (Years)	Race	BMI	Medical History	T1D Duration	AutoAb (GADA+, IA-2+, mIAA+, and/or ZnT8+)[A]	HbA1c	C-peptide (ng/mL)	Perifusion (Penn)	Perifusion (Vanderbilt)	Flow CyTOF	IMC	scRNAseq	pCREB Analysis
**Control Donors**																
HPAP-012	nPod	F	18	White	29.6	—	—	—	4.5	4.1	x	x	x	x		x
HPAP-018	Penn	M	31	Hispanic or Latino	24.5	—	—	—	5.4	6.40	x	x	x	x		x
HPAP-022	Penn	F	39	White	34.7	—	—	—	4.7	9.35			x		x	
HPAP-026	nPod	M	24	White	20.6	—	—	—	4.9	0.25	x	x	x		x	x
HPAP-027	Penn	F	31	White	32.7	—	—	—	4.4	7.06	x		x	x		x
HPAP-034	Penn	M	13	White	18.7	—	—	—	5.2	12.7			x		x	
HPAP-035	Penn	M	35	White	26.9	—	—	—	5.2	15.90	x	x	x	x	x	x
HPAP-036	nPod	F	23	White	16.0	—	—	—	5.2	1.12	x		x	x	x	x
HPAP-037	Penn	F	35	White	21.9	—	—	—	5.3	4.75	x		x		x	x
HPAP-039	nPod	F	5	White	16.3	—	—	—	6.8	1.88			x		x	
HPAP-040	Penn	M	35	White	24.0	—	—	—	5.4	7.01	x	x	x		x	
HPAP-046	Penn	M	19	Black or African American	21.0	—	—	—	5.7	20.74	x	x				
HPAP-047	Penn	M	8	White	16.8	—	—	—	ND	1.24			x		x	
HPAP-052	Penn	M	27	Black or African American	38.7	—	—	—	5.2	4.07	x	x	x			
HPAP-054	Penn	F	40	White	30.0	—	—	—	4.8	6.38	x	x	x			
HPAP-056	Penn	M	33	White	32.9	—	—	—	5.6	14.41	x	x	x			
HPAP-059	Penn	M	35	White	38.0	—	—	—	5.1	8.18	x	x	x			
HPAP-074	Penn	F	40	White	36.9	—	—	—	6.3	4.25	x		x			
HPAP-075	Penn	M	35	White	27.5	—	—	—	6.0	11.97	x		x			
HPAP-080	nPod	M	22	Black or African American	35.7	—	—	—	5.4	15.35	x	x	x			
ICRH91[B]	Penn	F	35	White	23.6	—	—	—	4.6	N/A	x			x		
ICRH99[C]	Penn	M	17	White	25.6	—	—	—	5.0	N/A	x			x		
ICRH100[D]	Penn	M	30	White	22.4	—	—	—	5.3	N/A	x			x		
**GADA+ Donors**																
HPAP-003	nPod	M	29	White	24.5	—	—	GADA+	5.6	9.00	x	x	x	x		x
HPAP-008	nPod	F	24	White	31.9	—	—	GADA+	5.2	27.05	x	x	x	x		x
HPAP-017	nPod	M	30	White	23.7	—	—	GADA+	5.5	3.71	x	x	x	x		x
HPAP-019	nPod	M	22	White	29.8	—	—	GADA+	5.2	8.82	x	x	x	x		x
HPAP-024	nPod	M	18	White	24.3	—	—	GADA+	5.5	5.6			x		x	
HPAP-029	nPod	M	23	White	28.6	—	—	GADA+	5.3	3.83	x	x	x	x	x	x
HPAP-038	nPod	M	13	White	18.3	—	—	GADA+	5.7	8.29	x	x	x	x	x	x
HPAP-045	nPod	F	27	White	26.2	—	—	GADA+	5.2	1.70	x	x	x		x	
HPAP-049	nPod	M	29	White	32.7	—	—	GADA+	5.4	6.15	x	x	x		x	
HPAP-050	nPod	F	22	Hispanic or Latino	29.0	—	—	GADA+	5.1	3.79	x		x		x	
**T1D Donors**																
HPAP-002	nPod	M	26	Hispanic or Latino	16.4	T1D	5 years	—	9.8	0.51	x		x			
HPAP-015	nPod	M	29	White	22.0	T1D	7 years	mIAA+	ND	0.03	x		x			
HPAP-020	nPod	M	14	White	13.3	T1D	0	GADA+, IA-2+, mIAA+, ZnT8+	ND	0.37	x		x			
HPAP-021	nPod	F	13	White	21.4	T1D	7 years	mIAA+	ND	<0.02	x		x			
HPAP-055	Penn	M	24	Hispanic or Latino	27.9	T1D	7 years	GADA+, IA-2+, mIAA+, ZnT8+	10.8	<0.02	x		x			
HPAP-071	nPod	F	12	White	15.4	T1D	3 years	IA-2+	9.8	0.06	x					

[A]All donors were tested for the presence of all four autoantibodies in the panel.
[B]ICRH91 (UNOS ADBD275)
[C]ICRH99 (UNOS ADGB379)
[D]ICRH100 (UNOS ADID386)

Supplemental Table 2. Differentially expressed genes between GADA+ and control alpha cells.

Gene Symbol	Description	log_2FC	padj
MRLN	myoregulin	-2.575	0.002
PSMB10	proteasome 20S subunit beta 10	-2.591	0.002
SAMD11	sterile alpha motif domain containing 11	-0.159	0.004
G6PC2	glucose-6-phosphatase catalytic subunit 2	-2.775	0.005
SCD5	stearoyl-CoA desaturase 5	-0.205	0.005
GPM6A	glycoprotein M6A	-0.081	0.005
TCIM	transcriptional and immune response regulator	-0.084	0.005
NPM3	nucleophosmin/nucleoplasmin 3	-1.702	0.005
PDX1	pancreatic and duodenal homeobox 1	-0.118	0.005
MRPL52	mitochondrial ribosomal protein L52	-1.497	0.005
SIX3-AS1	SIX3 antisense RNA 1	-0.078	0.008
PKIB	cAMP-dependent protein kinase inhibitor beta	-2.296	0.008
GADD45GIP1	GADD45G interacting protein 1	-0.752	0.010
NEIL2	nei like DNA glycosylase 2	-1.001	0.010
FFAR4	free fatty acid receptor 4	-0.094	0.010
C2orf76	chromosome 2 open reading frame 76	-2.329	0.011
DLK1	delta like non-canonical Notch ligand 1	-0.069	0.011
TMEM99	KRT10 antisense RNA 1	-0.222	0.011
SDHAF3	succinate dehydrogenase complex assembly factor 3	-1.848	0.011
PCDH7	protocadherin 7	-0.079	0.015
SEMA6A	semaphorin 6A	-0.153	0.020
SNCA	synuclein alpha	-1.379	0.021
MAFA	MAF bZIP transcription factor A	-0.149	0.021
TGFBR3	transforming growth factor beta receptor 3	-0.094	0.024
HIBADH	3-hydroxyisobutyrate dehydrogenase	-1.754	0.024
C11orf74	intraflagellar transport associated protein	-1.929	0.024
MRPL24	mitochondrial ribosomal protein L24	-1.516	0.024
SP110	SP110 nuclear body protein	-0.094	0.024
RTL8C	retrotransposon Gag like 8C	-1.547	0.024
PPP1R11	protein phosphatase 1 regulatory inhibitor subunit 11	-1.506	0.024
STX8	syntaxin 8	-1.069	0.024
ISOC1	isochorismatase domain containing 1	-1.570	0.027
TUBB2B	tubulin beta 2B class IIb	-0.165	0.027
MOSPD3	motile sperm domain containing 3	-0.909	0.028
CALD1	caldesmon 1	-0.118	0.028
TMEM126A	transmembrane protein 126A	-0.985	0.028
HSPBP1	HSPA (Hsp70) binding protein 1	-1.345	0.030
FAM229B	family with sequence similarity 229 member B	-1.054	0.030
TMEM126B	transmembrane protein 126B	-1.692	0.030
ZNF609	zinc finger protein 609	1.287	0.037
S100A11	S100 calcium binding protein A11	-1.412	0.040
AMN1	antagonist of mitotic exit network 1 homolog	-1.111	0.040
LINC02182	long intergenic non-protein coding RNA 2182	-0.177	0.040
PYCR2	pyrroline-5-carboxylate reductase 2	-1.587	0.040
RAB3C	RAB3C, member RAS oncogene family	-1.338	0.040
HMGN4	high mobility group nucleosomal binding domain 4	-1.846	0.043
CCND1	cyclin D1	-0.135	0.043
BORCS8	BLOC-1 related complex subunit 8	-1.480	0.043
CMSS1	cms1 ribosomal small subunit homolog	-1.601	0.044
TSHZ2	teashirt zinc finger homeobox 2	-0.091	0.044
SIX3	SIX homeobox 3	-0.099	0.044
MGST3	microsomal glutathione S-transferase 3	-0.687	0.045

Gene expression data obtained by scRNAseq was compared between alpha cells from GADA+ and control donors. Genes with adjusted p-value (padj)<0.05 from the DESeq2 analyses are listed here. Fold change (FC) compares GADA+ donors to controls.

HPAP Consortium

University of Pennsylvania
- Nicolai Doliba
- Erin Duffy
- Babak Faryabi
- Klaus H. Kaestner
- Chengyang Liu
- Elisabetta Manduchi
- Ali Naji
- Andrea Rozo
- Jonathan Schug
- Suzanne Shapira
- Doris Stoffers
- Golnaz Vahedi
- Benjamin Voight

Vanderbilt University
- Marcela Brissova
- Chunhua Dai
- Alvin C. Powers
- Diane Saunders

University of Florida
- Mark Atkinson
- Irina Kusmartseva
- Amanda Posgai
- Clive Wasserfall
- Mingder Yang

Stanford University
- Anna Gloyn
- Seung Kim

University of Alberta
- Patrick MacDonald

Mt. Sinai School of Medicine
- Dirk Homann
